# 2-[4,5-Bis(butyl­sulfan­yl)-1,3-dithiol-2-yl­idene]-5-methyl-5*H*-1,3-dithiolo[4,5-*c*]pyrrole-4-carbaldehyde

**DOI:** 10.1107/S160053681004910X

**Published:** 2010-11-30

**Authors:** Rui-Bin Hou, Bing-Zhu Yin

**Affiliations:** a Key Laboratory of Natural Resources of Changbai Mountain & Functional Molecules (Yanbian University), Ministry of Education, Yanji 133002, People’s Republic of China

## Abstract

In the title compound, C_18_H_23_NOS_6_, the dithiol­opyrrole ring is almost planar [r.m.s. deviation = 0.044 (3) Å] and makes a dihedral angle of 25.11 (7)° with the dithiole ring. In the crystal, pairs of neighboring mol­ecules are connected by weak inter­molecular C—H⋯O inter­actions. These dimers are further linked into a chain along [110] by C—H⋯O inter­actions.

## Related literature

For background to tetra­thia­fulvalenes, see: Jeppesen *et al.* (1999 ▶); Hansel *et al.* (2004 ▶). For the synthesis, see: An *et al.* (2009 ▶). For a related structure, see: Leng *et al.* (2009 ▶)
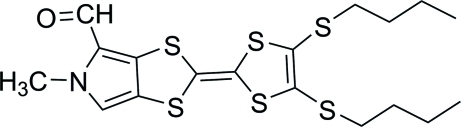


## Experimental

### 

#### Crystal data

C_18_H_23_NOS_6_
*M* *_r_* = 461.73
Triclinic, 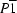

*a* = 7.4227 (15) Å
*b* = 8.8356 (18) Å
*c* = 17.811 (4) Å
α = 93.44 (3)°
β = 99.37 (3)°
γ = 105.31 (3)°
*V* = 1105.1 (4) Å^3^
*Z* = 2
Mo *K*α radiation
μ = 0.63 mm^−1^
*T* = 291 K
0.12 × 0.11 × 0.10 mm

#### Data collection

Rigaku R-AXIS RAPID diffractometer
Absorption correction: multi-scan (*ABSCOR*; Higashi, 1995 ▶) *T*_min_ = 0.929, *T*_max_ = 0.940
10707 measured reflections
4956 independent reflections
3298 reflections with *I* > 2σ(*I*)
*R*_int_ = 0.035

#### Refinement

*R*[*F* ^2^ > 2σ(*F* ^2^)] = 0.051
*wR*(*F* ^2^) = 0.176
*S* = 1.06
4956 reflections
238 parameters
H-atom parameters constrained
Δρ_max_ = 0.51 e Å^−3^
Δρ_min_ = −0.44 e Å^−3^

### 

Data collection: *RAPID-AUTO* (Rigaku, 1998 ▶); cell refinement: *RAPID-AUTO*; data reduction: *CrystalStructure* (Rigaku/MSC, 2002 ▶); program(s) used to solve structure: *SHELXS97* (Sheldrick, 2008 ▶); program(s) used to refine structure: *SHELXL97* (Sheldrick, 2008 ▶); molecular graphics: *PLATON* (Spek, 2009 ▶); software used to prepare material for publication: *SHELXL97*.

## Supplementary Material

Crystal structure: contains datablocks global, I. DOI: 10.1107/S160053681004910X/ng5074sup1.cif

Structure factors: contains datablocks I. DOI: 10.1107/S160053681004910X/ng5074Isup2.hkl

Additional supplementary materials:  crystallographic information; 3D view; checkCIF report

## Figures and Tables

**Table 1:** Hydrogen-bond geometry (Å, °)

*D*—H⋯*A*	*D*—H	H⋯*A*	*D*⋯*A*	*D*—H⋯*A*
C3—H3*B*⋯O1^i^	0.97	2.79	3.444 (5)	125
C4—H4*A*⋯O1^i^	0.97	2.71	3.368 (5)	126
C18—H18⋯O1^ii^	0.93	2.58	3.412 (5)	150

Symmetry codes: (i) 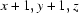; (ii) 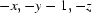.

**Fig. 1.**
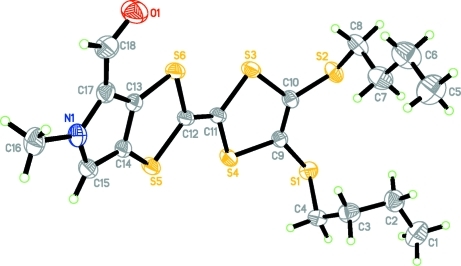
The asymmetric of title compound, with the atom numbering. Displacement ellipsoids of non-H atoms are drawn at the 30% probalility level.

## supplementary crystallographic information

### Comment

The tetrathiafulvalenes (TTFs) have become an interesting theme of organic synthesis (Jeppesen *et al.*, 1999). This is due to the high electrical conductivity and super conductor properties of these highly sophisticated compounds. Becher has recently synthesized a series novel donor-π-acceptor dyads based on the monopyrrolo-TTF (MPTTF), which exhibit good third-order non-linear optical properties (Hansel *et al.* 2004). In this paper, we report the crystal structure of the title compound, which is a key precursor of the dyads.

The title compound, as shown in Fig. 1, all bond lengths and angles are normal and comparable with those reported for the related structure (Leng *et al.*, 2009). In the title compound, the dithiolopyrrole ring and attached C16, C18 and O1 atoms are nearlly coplanar [mean deviation from the mean plane = 0.044 (3) Å. The dihedral angle between the dithiolopyrrole ring and dithiole ring is 25.11 (7) °. In the crystal, weak C—H···O hydrogen bonds (table 1) link the molecules into dimer firstly and the dimers are further linked to form one-dimensional chain along [a+b] direction.

### Experimental

The title compound was prepared according to literature (An *et al.*, 2009). Crystals suitable for single-crystal X-ray diffraction were grown by recrystallization from mixture of dichloromethane and petroleum (60–90 °C).

### Refinement

Carbon-bound H-atoms were placed in calculated positions with C—H = 0.93–0.97 A and were included in the refinement in the riding model with *U*_iso_(H) = 1.2 or 1.5 *U*_eq_(C).

### Crystal data

**Table:**

C_18_H_23_NOS_6_	*Z* = 2
*M**_r_* = 461.73	*F*(000) = 484
Triclinic, *P*1	*D*_x_ = 1.388 Mg m^−^^3^
Hall symbol: -P 1	Mo *K*α radiation, λ = 0.71073 Å
*a* = 7.4227 (15) Å	Cell parameters from 7048 reflections
*b* = 8.8356 (18) Å	θ = 3.2–27.4°
*c* = 17.811 (4) Å	µ = 0.63 mm^−^^1^
α = 93.44 (3)°	*T* = 291 K
β = 99.37 (3)°	Block, yellow
γ = 105.31 (3)°	0.12 × 0.11 × 0.10 mm
*V* = 1105.1 (4) Å^3^	

### Data collection

**Table:**

Rigaku R-AXIS RAPID diffractometer	4956 independent reflections
Radiation source: fine-focus sealed tube	3298 reflections with *I* > 2σ(*I*)
graphite	*R*_int_ = 0.035
ω scans	θ_max_ = 27.5°, θ_min_ = 3.2°
Absorption correction: multi-scan (*ABSCOR*; Higashi, 1995)	*h* = −9→9
*T*_min_ = 0.929, *T*_max_ = 0.940	*k* = −11→11
10707 measured reflections	*l* = −23→23

### Refinement

**Table:**

Refinement on *F*^2^	Primary atom site location: structure-invariant direct methods
Least-squares matrix: full	Secondary atom site location: difference Fourier map
*R*[*F*^2^ > 2σ(*F*^2^)] = 0.051	Hydrogen site location: inferred from neighbouring sites
*wR*(*F*^2^) = 0.176	H-atom parameters constrained
*S* = 1.06	*w* = 1/[σ^2^(*F*_o_^2^) + (0.103*P*)^2^] where *P* = (*F*_o_^2^ + 2*F*_c_^2^)/3
4956 reflections	(Δ/σ)_max_ = 0.001
238 parameters	Δρ_max_ = 0.51 e Å^−^^3^
0 restraints	Δρ_min_ = −0.44 e Å^−^^3^

### Special details

**Table:**

Geometry. All e.s.d.'s (except the e.s.d. in the dihedral angle between two l.s. planes) are estimated using the full covariance matrix. The cell e.s.d.'s are taken into account individually in the estimation of e.s.d.'s in distances, angles and torsion angles; correlations between e.s.d.'s in cell parameters are only used when they are defined by crystal symmetry. An approximate (isotropic) treatment of cell e.s.d.'s is used for estimating e.s.d.'s involving l.s. planes.
Refinement. Refinement of *F*^2^ against ALL reflections. The weighted *R*-factor *wR* and goodness of fit *S* are based on *F*^2^, conventional *R*-factors *R* are based on *F*, with *F* set to zero for negative *F*^2^. The threshold expression of *F*^2^ > σ(*F*^2^) is used only for calculating *R*-factors(gt) *etc*. and is not relevant to the choice of reflections for refinement. *R*-factors based on *F*^2^ are statistically about twice as large as those based on *F*, and *R*- factors based on ALL data will be even larger.

### Fractional atomic coordinates and isotropic or equivalent isotropic displacement parameters (Å^2^)

**Table:**

	*x*	*y*	*z*	*U*_iso_*/*U*_eq_	
C1	1.3588 (7)	0.8577 (6)	0.3605 (3)	0.0992 (16)	
H1A	1.4403	0.8569	0.3239	0.149*	
H1B	1.4309	0.8665	0.4113	0.149*	
H1C	1.3067	0.9459	0.3554	0.149*	
C2	1.2000 (6)	0.7069 (5)	0.3463 (2)	0.0746 (11)	
H2A	1.2529	0.6206	0.3598	0.090*	
H2B	1.1120	0.7139	0.3802	0.090*	
C3	1.0910 (6)	0.6676 (5)	0.2657 (2)	0.0685 (10)	
H3A	1.0039	0.5628	0.2612	0.082*	
H3B	1.1798	0.6642	0.2317	0.082*	
C4	0.9789 (5)	0.7804 (5)	0.2387 (2)	0.0612 (9)	
H4A	0.9227	0.7494	0.1851	0.073*	
H4B	1.0656	0.8853	0.2429	0.073*	
C5	1.0794 (8)	0.1668 (7)	0.4134 (3)	0.1074 (17)	
H5A	1.0622	0.1460	0.3588	0.161*	
H5B	1.1150	0.0815	0.4370	0.161*	
H5C	1.1778	0.2634	0.4305	0.161*	
C6	0.8994 (9)	0.1813 (7)	0.4347 (3)	0.1053 (17)	
H6A	0.7974	0.0886	0.4120	0.126*	
H6B	0.9111	0.1849	0.4899	0.126*	
C7	0.8498 (7)	0.3235 (6)	0.4094 (3)	0.0837 (13)	
H7A	0.8372	0.3194	0.3542	0.100*	
H7B	0.9524	0.4161	0.4317	0.100*	
C8	0.6652 (7)	0.3397 (5)	0.4319 (2)	0.0735 (11)	
H8A	0.6734	0.3350	0.4866	0.088*	
H8B	0.5604	0.2521	0.4059	0.088*	
C9	0.6295 (5)	0.6022 (4)	0.26157 (16)	0.0458 (7)	
C10	0.5642 (5)	0.4948 (4)	0.30804 (17)	0.0483 (7)	
C11	0.4100 (4)	0.3654 (3)	0.17027 (16)	0.0445 (7)	
C12	0.3512 (4)	0.2543 (3)	0.10934 (16)	0.0438 (7)	
C13	0.2413 (4)	−0.0068 (3)	0.02430 (17)	0.0440 (7)	
C14	0.3053 (4)	0.1071 (3)	−0.02319 (16)	0.0439 (7)	
C15	0.2900 (5)	0.0300 (4)	−0.09493 (18)	0.0525 (8)	
H15	0.3207	0.0776	−0.1381	0.063*	
C16	0.1719 (6)	−0.2460 (5)	−0.1579 (2)	0.0688 (10)	
H16A	0.0363	−0.2788	−0.1743	0.103*	
H16B	0.2154	−0.3354	−0.1440	0.103*	
H16C	0.2307	−0.2022	−0.1989	0.103*	
C17	0.1886 (5)	−0.1548 (4)	−0.01838 (19)	0.0510 (7)	
C18	0.1247 (6)	−0.3038 (4)	0.0080 (2)	0.0639 (9)	
H18	0.0960	−0.3934	−0.0267	0.077*	
N1	0.2228 (4)	−0.1257 (3)	−0.09119 (15)	0.0526 (7)	
O1	0.1051 (5)	−0.3211 (3)	0.07376 (16)	0.0820 (9)	
S1	0.79120 (13)	0.78869 (10)	0.29159 (5)	0.0561 (3)	
S2	0.61844 (16)	0.52311 (12)	0.40750 (5)	0.0659 (3)	
S3	0.38334 (14)	0.32527 (10)	0.26366 (5)	0.0567 (3)	
S4	0.52382 (14)	0.56342 (9)	0.16415 (4)	0.0536 (2)	
S5	0.37538 (13)	0.30352 (9)	0.01664 (4)	0.0524 (2)	
S6	0.24240 (13)	0.05437 (9)	0.11878 (4)	0.0516 (2)	

### Atomic displacement parameters (Å^2^)

**Table:**

	*U*^11^	*U*^22^	*U*^33^	*U*^12^	*U*^13^	*U*^23^
C1	0.068 (3)	0.109 (4)	0.115 (4)	0.023 (3)	0.011 (3)	−0.006 (3)
C2	0.074 (3)	0.087 (3)	0.069 (3)	0.035 (2)	0.011 (2)	0.010 (2)
C3	0.073 (3)	0.068 (2)	0.066 (2)	0.018 (2)	0.023 (2)	−0.0010 (19)
C4	0.061 (2)	0.070 (2)	0.0535 (19)	0.0135 (18)	0.0181 (17)	0.0137 (17)
C5	0.099 (4)	0.107 (4)	0.122 (5)	0.039 (3)	0.027 (3)	−0.005 (3)
C6	0.125 (5)	0.117 (4)	0.099 (4)	0.061 (4)	0.046 (3)	0.022 (3)
C7	0.103 (4)	0.083 (3)	0.072 (3)	0.031 (3)	0.027 (3)	0.009 (2)
C8	0.094 (3)	0.082 (3)	0.050 (2)	0.032 (2)	0.012 (2)	0.0188 (19)
C9	0.0491 (17)	0.0483 (16)	0.0380 (15)	0.0111 (14)	0.0081 (13)	−0.0007 (12)
C10	0.0561 (19)	0.0532 (17)	0.0366 (15)	0.0176 (15)	0.0089 (14)	0.0004 (13)
C11	0.0497 (18)	0.0417 (15)	0.0383 (15)	0.0053 (13)	0.0094 (13)	0.0053 (12)
C12	0.0484 (17)	0.0414 (14)	0.0378 (14)	0.0038 (13)	0.0107 (13)	0.0066 (12)
C13	0.0437 (16)	0.0421 (15)	0.0410 (15)	0.0058 (13)	0.0039 (13)	0.0028 (12)
C14	0.0460 (17)	0.0414 (15)	0.0388 (15)	0.0052 (13)	0.0042 (13)	0.0038 (12)
C15	0.060 (2)	0.0524 (18)	0.0425 (16)	0.0105 (16)	0.0079 (15)	0.0079 (14)
C16	0.072 (3)	0.064 (2)	0.061 (2)	0.0154 (19)	0.0018 (19)	−0.0205 (18)
C17	0.0520 (19)	0.0439 (16)	0.0504 (18)	0.0074 (14)	0.0010 (14)	0.0021 (13)
C18	0.072 (2)	0.0439 (17)	0.069 (2)	0.0075 (17)	0.0110 (19)	0.0014 (16)
N1	0.0560 (17)	0.0517 (15)	0.0452 (14)	0.0140 (13)	0.0012 (12)	−0.0056 (12)
O1	0.109 (2)	0.0570 (15)	0.0691 (18)	0.0038 (15)	0.0158 (17)	0.0148 (13)
S1	0.0593 (5)	0.0486 (4)	0.0552 (5)	0.0078 (4)	0.0121 (4)	−0.0066 (4)
S2	0.0881 (7)	0.0735 (6)	0.0349 (4)	0.0241 (5)	0.0069 (4)	0.0004 (4)
S3	0.0694 (6)	0.0540 (5)	0.0389 (4)	0.0006 (4)	0.0148 (4)	0.0056 (3)
S4	0.0736 (6)	0.0425 (4)	0.0383 (4)	0.0066 (4)	0.0071 (4)	0.0051 (3)
S5	0.0704 (6)	0.0412 (4)	0.0381 (4)	0.0023 (4)	0.0097 (4)	0.0066 (3)
S6	0.0623 (5)	0.0431 (4)	0.0422 (4)	−0.0001 (4)	0.0128 (4)	0.0074 (3)

### Geometric parameters (Å, °)

**Table:**

C1—C2	1.504 (6)	C9—C10	1.342 (5)
C1—H1A	0.9600	C9—S1	1.756 (3)
C1—H1B	0.9600	C9—S4	1.757 (3)
C1—H1C	0.9600	C10—S2	1.739 (3)
C2—C3	1.500 (5)	C10—S3	1.767 (3)
C2—H2A	0.9700	C11—C12	1.350 (4)
C2—H2B	0.9700	C11—S4	1.749 (3)
C3—C4	1.510 (5)	C11—S3	1.753 (3)
C3—H3A	0.9700	C12—S5	1.757 (3)
C3—H3B	0.9700	C12—S6	1.769 (3)
C4—S1	1.818 (3)	C13—C14	1.391 (4)
C4—H4A	0.9700	C13—C17	1.399 (4)
C4—H4B	0.9700	C13—S6	1.734 (3)
C5—C6	1.482 (7)	C14—C15	1.385 (4)
C5—H5A	0.9600	C14—S5	1.744 (3)
C5—H5B	0.9600	C15—N1	1.344 (4)
C5—H5C	0.9600	C15—H15	0.9300
C6—C7	1.475 (7)	C16—N1	1.475 (4)
C6—H6A	0.9700	C16—H16A	0.9600
C6—H6B	0.9700	C16—H16B	0.9600
C7—C8	1.528 (6)	C16—H16C	0.9600
C7—H7A	0.9700	C17—N1	1.387 (4)
C7—H7B	0.9700	C17—C18	1.413 (5)
C8—S2	1.807 (4)	C18—O1	1.217 (5)
C8—H8A	0.9700	C18—H18	0.9300
C8—H8B	0.9700		
			
C2—C1—H1A	109.5	C7—C8—H8B	109.3
C2—C1—H1B	109.5	S2—C8—H8B	109.3
H1A—C1—H1B	109.5	H8A—C8—H8B	107.9
C2—C1—H1C	109.5	C10—C9—S1	125.3 (2)
H1A—C1—H1C	109.5	C10—C9—S4	117.4 (2)
H1B—C1—H1C	109.5	S1—C9—S4	116.88 (18)
C3—C2—C1	115.1 (4)	C9—C10—S2	125.1 (3)
C3—C2—H2A	108.5	C9—C10—S3	116.0 (2)
C1—C2—H2A	108.5	S2—C10—S3	118.30 (19)
C3—C2—H2B	108.5	C12—C11—S4	123.3 (2)
C1—C2—H2B	108.5	C12—C11—S3	123.5 (2)
H2A—C2—H2B	107.5	S4—C11—S3	113.19 (16)
C2—C3—C4	115.4 (3)	C11—C12—S5	121.2 (2)
C2—C3—H3A	108.4	C11—C12—S6	121.7 (2)
C4—C3—H3A	108.4	S5—C12—S6	117.09 (16)
C2—C3—H3B	108.4	C14—C13—C17	108.2 (3)
C4—C3—H3B	108.4	C14—C13—S6	118.5 (2)
H3A—C3—H3B	107.5	C17—C13—S6	133.4 (3)
C3—C4—S1	114.2 (3)	C15—C14—C13	107.6 (3)
C3—C4—H4A	108.7	C15—C14—S5	135.4 (2)
S1—C4—H4A	108.7	C13—C14—S5	117.0 (2)
C3—C4—H4B	108.7	N1—C15—C14	107.9 (3)
S1—C4—H4B	108.7	N1—C15—H15	126.0
H4A—C4—H4B	107.6	C14—C15—H15	126.0
C6—C5—H5A	109.5	N1—C16—H16A	109.5
C6—C5—H5B	109.5	N1—C16—H16B	109.5
H5A—C5—H5B	109.5	H16A—C16—H16B	109.5
C6—C5—H5C	109.5	N1—C16—H16C	109.5
H5A—C5—H5C	109.5	H16A—C16—H16C	109.5
H5B—C5—H5C	109.5	H16B—C16—H16C	109.5
C7—C6—C5	112.5 (5)	N1—C17—C13	105.7 (3)
C7—C6—H6A	109.1	N1—C17—C18	126.9 (3)
C5—C6—H6A	109.1	C13—C17—C18	127.3 (3)
C7—C6—H6B	109.1	O1—C18—C17	123.6 (3)
C5—C6—H6B	109.1	O1—C18—H18	118.2
H6A—C6—H6B	107.8	C17—C18—H18	118.2
C6—C7—C8	112.7 (4)	C15—N1—C17	110.6 (3)
C6—C7—H7A	109.0	C15—N1—C16	124.0 (3)
C8—C7—H7A	109.0	C17—N1—C16	125.0 (3)
C6—C7—H7B	109.0	C9—S1—C4	101.26 (16)
C8—C7—H7B	109.0	C10—S2—C8	102.69 (17)
H7A—C7—H7B	107.8	C11—S3—C10	94.65 (15)
C7—C8—S2	111.8 (3)	C11—S4—C9	94.30 (15)
C7—C8—H8A	109.3	C14—S5—C12	93.61 (14)
S2—C8—H8A	109.3	C13—S6—C12	93.09 (14)

### Hydrogen-bond geometry (Å, °)

**Table:**

*D*—H···*A*	*D*—H	H···*A*	*D*···*A*	*D*—H···*A*
C3—H3B···O1^i^	0.97	2.79	3.444 (5)	125
C4—H4A···O1^i^	0.97	2.71	3.368 (5)	126
C18—H18···O1^ii^	0.93	2.58	3.412 (5)	150

Symmetry codes: (i) *x*+1, *y*+1, *z*; (ii) −*x*, −*y*−1, −*z*.
